# Annotations

**Published:** 1904-01-16

**Authors:** 


### Annotations.

### St. Bartholomew's Hospital.

The position at the moment is remarkable. An attempt is being made to commit Bart's. to the issue of an appeal on January 26th, and the medical staff to the acceptance beforehand of some hurriedly prepared plans. In fact, it is not possible to prepare a scheme and organisation with plans before the 26th inst. which can hope to succeed in producing the half-million of money required The preparation of a perfected plan, in conjunction with the medical staff, must take two or three months at the least, and we cannot believe that the medical staff will consent to accept any plan until they have worked out every detail so as to secure that in all respects it shall be as comprehensive and complete as possible. Unfortunately there seem to be few people connected with St. Bartholomew's Hospital who understand the requirements which have to be met before an appeal for a large sum of money can be successfully made to the public in these days To contend on the one hand that the future of St. Bartholomew's Hospital is of great importance to the City of London, to the sick poor, and to the cause of medical education, in justification of an appeal for half a million of money, and, on the other, to render that appeal abortive by asking for contributions without submitting a perfected scheme, is but to court defeat in advance. We hope, therefore, the Governors will go to the Mansion House meeting with a proposal to leave the date when the appeal shall be issued to the discretion of the Appeal Committee, such date to be fixed after an unimpeachable plan prepared by the Building Committee has been approved by all the interests which have to be met in the matter. If this business-like step be taken the prospects of the appeal should be good, but otherwise we feel confident it must prove a fiasco. The responsibility for failure in the circumstances must, therefore, rest upon those authorities of St. Bartholomew's Hospital who ignore the teachings of experience and the necessity for business methods in appeals of this kind in the present day.

### The Future of Electro-Therapeutics.

Physical methods of treating disease, like many other therapeutic devices, have had a somewhat chequered career. At one time they have enjoyed a large measure of professional repute, at others they have been neglected or despised. The hot fit, as is natural, is invariably succeeded by the stage of reaction. The explanation of this is, in part at least, to be found in the ungoverned enthusiasm which is with rare exceptions the mark of those who practise some special form of treatment. So much is claimed and professed whilst so little stands the test of experience that discredit falls upon the cause as a whole until, in course of time, there arises some new advocate whose personality or methods once more attract disciples. Such a course of events has been conspicuously illustrated in the application of electricity to the treatment of disease, and hence it is not surprising to find that in the opinion of many history will repeat itself and the present-day activity prove to be a temporary and evanescent phenomenon. There are, however, reasons for hoping that, whatever be the real value of electricity as a therapeutic agent, this will ere long be defined in definite scientific terms. One ground for this anticipation is the foundation of the British Electro-Therapeutic Society which, as its name suggests, is devoted to the study of electricity in its curative aspects. No doubt such a society must include among its members some whose associations prevent the mental detachment necessary to clear and impartial vision. But the critic and sceptic may also count upon a hearing. In the admirable address recently delivered to the Society by Sir Isambard Owen, the collective responsibility of the members was presented with much point and force. It is undoubtedly true that the profession is greatly in need of sober guidance, and of a source of authoritative information on this matter. If the dangers of unwarranted pretension on the one hand and of incredulous neglect on the other are to be avoided some trustworthy voice must be supplied. This is the opportunity of the new society and we shall be much disappointed if it is not realized.

### Alcohol and Evolution.

Dr. Harry Campbell discusses this subject as bearing upon the possible influence exerted by alcohol in the evolution of the human race. Observing that none of the pre-agricultural peoples now living were acquainted with alcohol before they came into contact with more advanced races he argues that we may therefore conclude that man had learned to cultivate the vegetable kingdom for food before he began to prepare and use alcohol as a beverage. The date of the origin of agriculture is a matter of conjecture. But assuming that such ancient civilisations as those of Babylon and Egypt date back 30,000 years and that a similar period of time elapsed whilst the precursors of these peoples lived as migratory tribes cultivating the soil as they moved from place to place, it may be suggested that the practice of agriculture extends backwards over 60 millenniums. There is reason to believe that the use of alcohol as a beverage does not extend to more than 10,000 years, but even allowing a much longer period the event must be placed relatively late in man's evolutionary history. Hence the conclusion " that man had come well within reach of the highest rung of his long evolutionary ladder before he had felt the stimulus of that most subtle and potent fluid " This is a very different proposition from that advanced by a well-known medical writer some two or three years ago. Here the suggestion was that alcohol had been a chief aid in the development of the human brain, and that it was the discovery of fermentation which gave the ape-man his first impetus in the career of civilisation. Dr. Campbell's suggestion, even if adopted, leaves the affair a mystery ; for the purpose of his argument there was no need to advance further, but this has to be remembered, that the more useless or pernicious the action of alcohol is proved to be the more pertinent is the claim for an explanation of its use.

